# Influence of dietary patterns in the pathophysiology of Huntington's Disease: A literature review

**DOI:** 10.3934/Neuroscience.2024005

**Published:** 2024-04-12

**Authors:** Ubaid Ansari, Dawnica Nadora, Meraj Alam, Jimmy Wen, Shaheryar Asad, Forshing Lui

**Affiliations:** California Northstate University College of Medicine, USA

**Keywords:** Huntington's disease, neurodegenerative disease, mediterranean diet, vegan diet, carnivore diet, paleolithic diet, ketogenic diet

## Abstract

Huntington's disease (HD), a rare autosomal dominant neurodegenerative disease, causes the gradual deterioration of neurons in the basal ganglia, specifically in the striatum. HD displays a wide range of symptoms, from motor disturbances such as chorea, dystonia, and bradykinesia to more debilitating symptoms such as cognitive decline, behavioral abnormalities, and psychiatric disturbances. Current research suggests the potential use of dietary interventions as viable strategies for slowing the progression of HD. Most notably, the Mediterranean, vegan, carnivore, paleo, and ketogenic diets have gained attention due to their hypothesized impact on neuroprotection and symptomatic modulation in various neurodegenerative disorders. Despite substantial nutritional differences among these diets, they share a fundamental premise—that dietary factors have an influential impact in modifying pertinent biological pathways linked to neurodegeneration. Understanding the intricate interactions between these dietary regimens and HD pathogenesis could open avenues for personalized interventions tailored to the individual's specific needs and genetic background. Ultimately, elucidating the multifaceted effects of these diets on HD offers a promising framework for developing comprehensive therapeutic approaches that integrate dietary strategies with conventional treatments.

## 1. Introduction

Huntington's disease (HD) is a rare autosomal dominant neurodegenerative disorder that causes neurons in the basal ganglia, particularly in the striatum, to gradually break down and die [1]. Impairment of mitochondrial function, heightened oxidative stress, and increased neuronal excitotoxicity are all probable factors that contribute to the demise of striatal cells observed in this particular inherited disorder [2]. HD stems from a mutation within the huntingtin (HTT) gene on chromosome 4p, which is responsible for encoding the huntingtin (HTT) protein. This mutation results in an abnormal trinucleotide repeat expansion of cytosine, adenine, and guanine (CAG) in the HTT gene, causing an abnormally long polyglutamine (polyQ) expansion in the HTT protein, thereby exceeding the typical number of 10–35 repetitions [3]. The extent of the expansion correlates with the severity of the disease, where higher counts of CAG repetitions accelerate the onset of symptoms [1]. Manifestations of HD encompass motor irregularities such as chorea, dystonia, and bradykinesia, alongside cognitive decline, behavioral irregularities, and psychiatric disturbances [4]. As the search for effective treatments continues, emerging research suggests that dietary interventions might offer promising avenues to manage the progression of HD. Among these dietary regimens, the Mediterranean, vegan, carnivore, paleo, and ketogenic diets have garnered attention for their potential impact on neuroprotection and symptom modulation in various neurodegenerative conditions.

The Mediterranean diet, renowned for its emphasis on fruits, vegetables, whole grains, olive oil, and moderate consumption of fish and poultry, stands out for its proposed neuroprotective properties due to its rich array of antioxidants and anti-inflammatory compounds [5]. Conversely, the vegan diet, which is entirely plant-based and devoid of animal products, has gained recognition for its to reduce oxidative stress and inflammation, which are factors implicated in the progression of neurodegenerative diseases like HD [6]. In stark contrast, the carnivore diet, characterized by an exclusive consumption of animal-derived foods, challenges conventional nutritional wisdom, though it merits examination for its radical departure from conventional dietary norms and its potential effects on neuroinflammation and metabolic pathways [7]. Meanwhile, the paleo diet, inspired by the presumed dietary habits of our Paleolithic ancestors, focuses on lean meats, fish, fruits, vegetables, nuts, and seeds, aiming to optimize an individual's health by avoiding processed foods and grains [8]. Lastly, the ketogenic diet, characterized by a high fat and low carbohydrate intake, has shown promise in various neurological conditions due to its ability to induce ketosis, thus altering the brain's metabolism and potentially offering neuroprotective effects in HD [9].

While these diets significantly diverge in their nutritional compositions, they share an underlying premise—that dietary factors play a crucial role in modulating biological pathways relevant to neurodegeneration. This review aims to elucidate their potential mechanisms of action, the comparative effectiveness, and implications for clinical practice by comprehensively examining the current body of literature surrounding each diet's impact on HD. Understanding the nuanced effects of these diets on HD could provide valuable insights into strategies that manage this complex neurological disorder.

## 2. Review

### 2.1. Mediterranean Diet

The Mediterranean diet (MedD) is mainly characterized by the intake of fish and olive oil as the primary source of monounsaturated fats, as well as plant foods such as fruits, nuts, legumes, and cereals [10]. Additionally, it involves a limited intake of red meat, poultry, and dairy products, and is widely recognized for its health benefits due to its protective effects against various chronic diseases [10]. The protective effects of this diet, specifically through antioxidants like flavonoids, can combat free radical damage by reducing the concentration of reactive oxygen species like α-tocopherol radicals, activating antioxidant enzymes, and inhibiting certain oxidases such as xanthine oxidase, cyclooxygenase, lipoxygenase, and phosphoinositide 3-kinase [11]. Thus, integrating nutritional interventions such as the Mediterranean diet may either delay the onset or slow down the progression of neurodegenerative diseases such as HD.

MedD's potential to function as a neuroprotective agent against HD is due to its increased antioxidant effect and its ability to reduce inflammation. Olive oil, a major component of this diet, has been previously shown to be neuroprotective against oxidative stress by reducing the production of nitric oxide [12]. Moreover, in a study that investigated the efficacy of extra-virgin olive oil (EVOO) as a brain antioxidant that combated oxidative stress induced by 3-nitropropionic acid (3NP), the findings indicated that EVOO could mitigate the impact of free radicals triggered by 3NP [13]. In this study, 3NP was intraperitoneally injected into Wistar rats for four days to induce HD-like symptoms, which resulted in increased lipid peroxides levels and reduced glutathione content. However, upon administration of EVOO and hydroxytyrosol for 14 days, results showed a reduction in the lipid peroxidation product levels and a decrease in glutathione depletion in both the striatum and the rest of the brain [13]. This suggests that EVOO, which is found in MedD's, serves as a potent antioxidant against neurodegenerative diseases.

In addition, MedD's include a high consumption of sea fish such as mackerel or salmon on a regular basis. Sea fish has been identified to be a reliable source of both eicosapentaenoic acid (EPA) and docosahexaenoic acid (DHA), which are both not synthesized by human enzymes and must therefore be consumed via diet [14]. Although its exact mechanism is unknown, ethyl-EPA can improve neuronal dysfunction and stabilize mitochondrial integrity, and its supplementation may be beneficial for those diagnosed with HD [15]. In a 2005 animal study, Van Raamsdonk et al. examined the efficacy of ethyl-EPA treatment in a yeast artificial chromosome 128 (YAC128) mouse model of HD [16]. They reported that oral supplementation of ethyl-EPA to symptomatic mice resulted in a significant improvement of motor dysfunction. However, there was no observed improvement in the neurodegeneration of the YAC128 mouse model. Furthermore, a 2005 double-blind, randomized, placebo-controlled clinical trial did not show ethyl-EPA as an effective treatment for HD patients. In this study, a total of 135 HD patients were randomly assigned to either the treatment group (2 g/d ethyl-EPA) or the placebo group to test the efficacy of ethyl-EPA in attenuating HD symptoms. Despite the study outcome, an exploratory analysis revealed that a significantly higher number of participants in the ethyl-EPA treatment group displayed either a stable or an improved motor function [17]. Additionally, in a 2013 prospective cohort study, Marder et al. reported that MedD's were not associated with HD phenoconversion but observed that the high intake of dairy products had a two-fold increased risk in phenoconversion [18]. They noted that participants with a lower adherence to a MedD were correlated with an expanded CAG repeat length (CAG ≥ 37) and were marginally associated with risk for phenoconversion, which may suggest that the MedD diet can be a diet modification that can hinder the worsening of HD symptoms.

As a nutritional intervention for HD, the Mediterranean diet warrants further investigation as several studies have demonstrated conflicting evidence with HD onset, its severity, and its association with MedD consumption. In a 2009 randomized, controlled trial of ethyl-eicosapentaenoic acid in patients with HD, it was shown that the treatment was not beneficial during the 6 months of placebo-controlled evaluation of 316 HD patients [19]. There were no differences identified in the assessments of function, cognition, and global impression in the ethyl-EPA assignment group as compared to the participants in the placebo group. The six-month treatment duration might be insufficient to expect significant changes in the context of a dietary intervention [19]. Thus, new studies in the field are needed to validate the association between the consumption of certain diets such as the Mediterranean diet, with its nutritional components, and the symptom severity of HD.

### 2.2. Vegan Diet

The vegan diet omits all foods that come from animal sources and from animal by-products. There are several different forms of veganism that exist; however, all forms of veganism do not allow for the consumption of any form of meat or products that come from animal sources [20]. Despite this strict adherence to avoiding meat and food derived from animals, the Academy of Nutrition and Dietetics has stated that planned vegan diets are healthful, nutritionally adequate, and may even provide health benefits for the prevention and treatment of certain diseases [21]. A typical vegan diet often consists of a high intake of vegetables, fruits, whole grains, legumes, soy products, nuts, and seeds, all of which are rich in fibers and phytochemicals. Additionally, a vegan diet often consists of a low intake of saturated fats, which leads to the production of lower total and low-density lipoprotein (LDL) cholesterol levels and an improved serum glucose control [21]. However, despite the benefits of a vegan diet, it is critical for vegans to have a reliable source of vitamin B-12, such as through the intake of supplements [21]. Despite the fact that research is still being conducted to discover the exact mechanism through which a vegan diet reduces chronic disease, several meta-analysis studies have linked the foods consumed in a vegan diet with a reduction in chronic disease development [21]. A meta-analysis by Helmer et al. showed that a 200-g/d increase in fruit and vegetable consumption was associated with a 16% lower risk of stroke, an 8% lower risk of cardiovascular disease (CVD), a 3% lower risk of total cancer, and a 10% lower risk of all-cause mortality [22]. Furthermore, there was a strong dose-response relationship between consuming whole-grains and reduced total and cause-specific mortalities [22]. Additionally, there was an association between the legume intake and a lower risk of ischemic heart disease [22]. Taking all of these factors of a vegan diet together contributes to the reduction of chronic disease development, which is evident amongst individuals on a vegan diet.

One of the primary advantages of a vegan diet is that it produces an abundant number of antioxidants, which can effectively help combat any harm that is caused to cells through oxidative stress [23]. Antioxidants function by donating electrons to disrupt the chain reaction of oxidation reactions that can cause cellular damage [24]. Specifically, with a vegan diet, vitamins C and E, polyphenols, and carotenoids all significantly contribute as diverse antioxidants that can synergistically collaborate to combat the development of reactive oxygen species (ROS) that arise within cells. A vegan diet can significantly improve the health of mitochondria in cells where a large number of antioxidants are produced, thereby decreasing oxidative stress [23],[24].

Compelling data has shown that oxidative stress plays a critical role in the pathogenesis of neurodegenerative diseases such as HD, which is caused by a polyglutamine expansion in the H protein [25]. It has been shown that the mutant HTT (mHTT) proteins serve as a significant source of ROS due to the significant number of oxidized proteins in partially purified aggregates of mHTT proteins [26]. The large number of antioxidants produced from a vegan diet can serve to help reduce a significant amount of the oxidative stress caused by the mHTT proteins that are implicated in the pathogenesis of HD. Furthermore, HD has been linked to an increase in inflammatory markers such as interleukin-6 (IL-6), interleukin-10 (IL-10), C-reactive protein (CRP), C3, interferon-γ (IFN-γ), interleukin-1 (IL-1), interleukin-2 (IL-2), interleukin-8 (IL-8), and tumor necrosis factor-α (TNF-α) [27]. Since a vegan diet often consists of a high number of plant-based foods, these have been shown to significantly reduce the levels of CRP in individuals compared to other diets [28]. The other inflammatory markers are not significantly reduced in a vegan diet.

A considerable amount of evidence points to the fact that a vegan diet can help reduce ROS and improve the overall health of a cell by helping mitigate cell damage. However, despite these promising findings, further research is needed to fully determine the relationship between a vegan diet and HD. There is encouraging evidence that a vegan diet does decrease inflammation overall, which is often used as a biomarker to diagnose HD. However, further research is needed to fully determine the true impact a vegan diet has on the onset of HD.

### 2.3. Carnivore Diet

The carnivore diet shares a similar macromolecule composition as the ketogenic diet, with a minimal intake of carbohydrates. It differs slightly through the high consumption of animal products and saturated fats; however, a recent 2020 study has suggested that adequate essential nutrients can be obtained through this diet [29]. As a result, the low carbohydrate content stimulates the production of ketone bodies, which can be used as an alternative energy source for neuron and muscle cells. Ketosis has been shown to increase Peroxisome proliferator-activated receptor-y-coactivator-1a (PGC-1a) and mitochondrial remodeling, thus theoretically mitigating the mitochondrial decline in HD [30]. However, the carnivore diet, and other extreme implementations of the ketogenic diet by extension, typically include little to no plant-based foods. Of the plant-based foods, non-starchy vegetables are not only an important consideration for micronutrient intake and prebiotic health that is needed for energy metabolism, but also for the downstream effects of fiber breakdown products by the gut microbiome. Short-chain fatty acids that are produced support both ketogenic and mitochondrial cellular functions [31],[32]. In addition, ketones provide neuroprotection at the mitochondrial level via decreasing the number of free radicals and have been demonstrated to be neuroprotective in several neurodegenerative disorders such as Parkinson's, Alzheimer's, and Amyotrophic Lateral Sclerosis (ALS) [33]. Therefore, the lack of such nutrients may not be the most optimal in mitigating the effects of HD.

Mitochondrial function has been increasingly implicated in the pathogenesis of HD, with PGC-1a, neuron mitochondria number and stability, and complexes I, II, III, and IV of the mitochondrial electron transport chain being decreased and/or defective. The electron chain complexes are speculated to affect the basal ganglia specifically. The decreased mitochondrial function leads to an energy shortage that negatively impacts metabolically active cells, especially neuron and muscle cells [34]–[36]. Interestingly, other trinucleotide repeat expansion diseases share a common mechanism of mitochondrial impairment towards their pathogenesis [34].

Decreased lifespans in degenerative diseases, such as HD, have been implicated in response to the lowered consumption of fruit, vegetables, nuts, seeds, and whole grains. However, it is also connected with high sodium consumption levels [37]. In addition, increased appetite and weight loss is also associated with the pathogenesis of HD; thus, dietary intake has increasingly been investigated for HD patients [38]). Increased caloric intake in patients is associated with an increased chance of containing a greater number of CAG repeats (>37), a greater risk for disease development, and an earlier onset of HD. This may be as a compensatory mechanism to maintain body weight and energy homeostasis in the early stages of the disease [18],[39].

HD in mice and humans is associated with urea cycle deficiencies, which are characterized by increased blood citrulline and ammonia levels. In mice studies, a low protein diet has been shown to have beneficial effects by reducing hyperammonemia and resulting in improved urea cycle function, which ultimately led to improved motor skills [40]. In humans, Chen et al. investigated the effect of a high dietary protein intake, accounting for 26.3% of total calories over for six days, which did not exacerbate the urea cycle dysfunction observed in HD patients [38]. HD patients have a relatively high prevalence of low carnitine plasma levels, and carnitine supplementation demonstrated motor, cognitive, and behavioral improvements [41]. It is found in several animal products, is a lipid metabolism regulator that assists in beta-oxidation for energy production, and functions as an antioxidant. Therefore, the carnivore diet may serve to ameliorate motor pathway symptoms via an increased carnitine intake [42].

Animal products, particularly fatty fish, contain high amounts of omega-3 fatty acids (FAs). Meat contains significantly less omega-3 FAs, though white meat contains even less than red meat. Grass-fed meat contains a higher concentration of omega-3 than concentrate-fed counterparts and may serve as a suitable alternative for essential FA intake with the carnivore diet [43],[44]. The two predominant omega-3 FAs, eicosapentaenoic acid (EPA) and docosahexaenoic acid (DHA), have recently been discovered to produce lipid mediators named resolvins and neuroprotectins, respectively. These compounds decrease inflammation and oxidative stress and most noticeably exert its effects in the central nervous system. As neurodegeneration is an inflammatory process, there has been increased interest in exploring these compounds for their role in neuroprotection [15]. As HD is characterized by a disrupted lipid metabolism and insulin resistance, additional benefits of EPA and DHA for HD patients are their ability to downregulate lipid synthesis, increase mitochondrial turnover, and increase beta-oxidation [45]. Additionally, these lipid metabolism effects provide a reduced risk for CVD, which is a leading cause of death in HD patients, thus providing a potential improvement in clinical outcomes [15],[45]. The carnivore diet's minimal carbohydrate, ketogenesis, and omega-3 fatty acid consumption have been shown to modulate neuroprotection and mitochondrial function in vivo and in vitro, and thus should be investigated for its direct effects on HD pathogenesis.

### 2.4. Paleo Diet

The Paleolithic diet, better known as the Paleo diet, emulates the dietary patterns of ancient hunter-gatherer societies, particularly in the Paleolithic Era. The primary components of this diet are its exclusion of excess carbohydrates and an emphasis on lean meats and fish alongside the addition of seeds, nuts, and select vegetables that were common in this era [46]. In the modern implementation of the Paleo diet, there is a hallmark exclusion of dairy products, refined fats, excess sugars, and processed foods, which emulates the conditions faced by ancestral humans in the Paleolithic era [46]. It is thought that the adoption of the Paleo diet hinges on the notion that shifts in human dietary habits might have contributed to an increase in chronic conditions stemming from nutritional deficiencies. Consequently, some proponents suggest that the Paleo diet could potentially serve as a mitigating factor in the onset or progression of such chronic conditions through the exclusion of highly processed foods that can promote inflammation [47].

Within the context of HD, the Paleolithic diet can offer some beneficial effects by removing certain dietary components that may exacerbate the disease, most notably gluten [48]. The accumulation of misfolded huntingtin proteins in the brain is a classic feature of HD. Inflammatory responses to gluten can lead to an increase in tissue transglutaminase (TTG), which is a calcium-dependent ubiquitous enzyme that catalyzes the posttranslational modifications of proteins and is released from cells during inflammation [48]. One of the posttranslational modifications associated with TTG is the creation of bonds between amino acids such as glutamine. The expanded polyglutamine that is encoded by the CAG repeats could be a potential target of TTG activity, which could suggest a potential increase in the accumulation of misfolded huntingtin protein [49]. While this link may point towards a potential slowing of the disease, the effectiveness of dietary changes and the role of TTG in HD progression still requires further study.

HD has also been associated with increased inflammatory biomarkers, particularly IL-6 and IL-10, compared to other inflammatory markers (i.e., CRP, C3, IFN-γ, IL-1, IL-2, IL-8, and TNF-α) as indicated in a meta-analysis conducted by [27]. While the exact reason behind increased IL-6 levels is not fully known, it is significantly increased through all stages of disease progression and has the potential to be used as a biomarker for HD [50]. The Paleolithic diet has been shown to decrease levels of serum IL-6 in postmenopausal women, which could point toward another potential link between the diet and HD [53]. The exact mechanism behind this decrease needs additional study; however, certain nutrients such as omega-3 within the diet help contribute to an overall reduction in inflammation, which can potentially include IL-6. Omega-3 FA is one such nutrient, since the paleo diet is rich in fish and lean meats that contain omega-3s [51]. In normal cells, oxidized linoleic acids (omega-6), which are found in seed oils, can activate Nuclear Factor-Kappa Beta (NF-kB). NF-kB is a transcription factor that can promote inflammation and has been linked with increased IL-6 levels [51],[52]. Consumption of omega-3 along with a reduction of seed oil consumption in the Paleo diet can help stabilize the omega-6/omega-3 ratio, which can reduce the inflammation caused by linoleic acids, which in turn can potentially decrease IL-6 levels [54]. However, further studies need to be conducted on HD patients to determine whether the Paleo diet has these effects on IL-6 levels in HD patients and whether decreased IL-6 levels have a significant impact on the pathogenesis of HD [50].

### 2.5. Ketogenic Diet

The ketogenic diet, commonly known as the keto diet, is primarily characterized by a high intake of fat, a moderate consumption of protein, and a very low carbohydrate intake. This ratio is crucial to reach ketosis in order for carbohydrates to reach extremely low levels, thus forcing the body to use fat metabolism byproducts such as ketones in the liver as an alternative source of energy [55]. High levels of ketones can be maintained by continuously depriving the body of carbohydrates, thus allowing ketones to be used as an energy source by organs such as the brain without altering the blood pH [56]. This diet is different from “low-carbohydrate” diets as this specifically limits carbohydrates into less than 50 grams as compared to 130 grams per day [55]. In addition, a restricted carbohydrate diet is not sufficient to induce ketosis, since a higher intake of fats and proteins are needed to replace carbohydrates as a primary fuel source.

By restricting the caloric intake, the ketogenic diet may exhibit neuroprotective effects by enhancing mitochondrial functions, thus leading to a decrease in the production of ROS and an increase in the energy output [58]. This results in a reduction of inflammatory and pro-apoptotic activities, along with elevated levels of neuroprotective factors generated by ketone bodies, specifically β-hydroxybutyrate, which is capable of protecting neurons in culture against defects in mitochondrial energy production, as observed in both Alzheimer's disease and Parkinson's disease [58],[59]. The ketogenic diet has been shown to provide neuroprotective effects against several neurological diseases such as refractory epilepsy, Parkinson's disease, Alzheimer's disease, and traumatic brain injury, specifically in rodent models in which ketogenic diet implementation was able to reduce seizures in the mice by increasing the activation of adenosine A1 receptors [58], [60]. In a 2011 mouse model study conducted by Ruskin et al., transgenic mouse models of HD who were fed a ketogenic diet did not experience adverse effects in locomotor activity, coordination, and their working memory [59]. There were no significant changes in their lifespans; however, it is crucial to note that their weight loss was delayed. A common presentation of HD is progressive weight loss, and a slower progression of the disease has been linked with a high body weight; thus, it is crucial for HD patients to maintain their weight [61]. In the mouse model study, ketogenic diet intervention showed a delay in body weight loss in the mice models, even though this diet is widely known to result in weight loss [60]. Thus, the ketogenic diet was able to confer these benefits without exerting any negative effects on locomotor activity and memory.

Interestingly, a 2022 case study on a 41-year-old patient with progressive, deteriorating HD, revealed that management with a time-restricted ketogenic diet led to improvements in their motor symptoms, their daily living activities, their HD-related behavioral problems, and their mood-related quality of life [61]. It is worthy to note that there was a 52% improvement in the motor symptoms within this case study using the Unified HD Rating Scale (UHDRS®) Total Motor Score. Phillips et al. believed that this was the first documented case of incorporating either fasting or a ketogenic diet that resulted in improved motor symptoms in a hyperkinetic movement disorder [61]. They compared this change with tetrabenazine, which was the only available treatment indicated for chorea associated with HD, which improved baseline chorea by 23.5% with many adverse side effects, thus limiting its clinical use [61]. Moreover, the case study is also unique in that it led to a 50% to 100% improvement in terms of behavioral problems related to apathy, disorientation, anger, and irritability [61]. While these results were specific to a particular patient, the findings are promising, thus highlighting the necessity for additional research on metabolic strategies in HD.

As with any nutrition modification, adverse reactions must be considered. The current literature regarding the relationship between the ketogenic diet, insulin resistance, and nonalcoholic fatty liver disease (NAFLD) is complex and varies among individuals. However, the ketogenic diet has been associated with insulin resistance, NAFLD, and an increased risk of type 2 diabetes in rodents, although it was reported that these side effects were temporary [62]. Moreover, there is a lack of research on the effects of the ketogenic diet in HD; thus, further studies are warranted, especially in determining the long-term effects of the ketogenic diet on the symptoms and the course of this neurodegenerative disease.

## 3. Conclusion

HD is a complex autosomal dominant genetic disease, in which effective treatments are still subjects of ongoing research. Recent studies have shown that diet has a potential role to play in reducing the progression of HD. These diets have been shown to have a slight alleviating effect on the pathogenesis of several other neurological disorders such as ALS, dementia, Parkinson's, and Alzheimer's. This review examined several popular dietary patterns and their possible effects on the onset or progression of HD. Although these diets varied in terms of their macromolecule composition, they may have similarities in their neuroprotective effects due to the anti-inflammatory or antioxidant properties of these diets and how they could help reduce these stress factors in the pathogenesis of HD. It should be noted that the potential benefits of each diet and their composition were varied based on the different studies that were included in this review. Some of the variables that could alter the outcome of these diets included lifestyle, genetic susceptibility, and the environment, along with the interactions between these different variables. Determining the beneficial or harmful mechanisms of certain foods can elicit a better understanding of the pathogenesis of HD and how these foods can affect the progression of the disease. The limitations of this study are the significant heterogeneity of the studies examined. There is currently no standardized approach to evaluate dietary impact and control for the variables that can impact each study's results. Due to various views regarding the benefits of these diets within the literature, future studies on the topic should be conducted through randomized controlled trials (RCTs) with relevant patient populations and standardized techniques to properly evaluate the effects of these diets discussed in this study on HD.
